# Venous Graft-Derived Cells Participate in Peripheral Nerve Regeneration

**DOI:** 10.1371/journal.pone.0024801

**Published:** 2011-09-23

**Authors:** Mitra Lavasani, Sebastian Gehrmann, Burhan Gharaibeh, Katherine A. Clark, Robert A. Kaufmann, Bruno Péault, Robert J. Goitz, Johnny Huard

**Affiliations:** 1 Stem Cell Research Center, Children's Hospital of Pittsburgh of UPMC, Pittsburgh, Pennsylvania, United States of America; 2 Department of Bioengineering, University of Pittsburgh, Pittsburgh, Pennsylvania, United States of America; 3 Department of Orthopaedic Surgery, University of Pittsburgh Medical Center, Pittsburgh, Pennsylvania, United States of America; 4 Center for Biologic Imaging, Departments of Cell Biology and Physiology, University of Pittsburgh School of Medicine, Pittsburgh, Pennsylvania, United States of America; 5 Department of Pediatrics, University of Pittsburgh, Pittsburgh, Pennsylvania, United States of America; 6 Department of Molecular Genetics and Biochemistry, University of Pittsburgh, Pittsburgh, Pennsylvania, United States of America; University of California Merced, United States of America

## Abstract

**Background:**

Based on growing evidence that some adult multipotent cells necessary for tissue regeneration reside in the walls of blood vessels and the clinical success of vein wrapping for functional repair of nerve damage, we hypothesized that the repair of nerves via vein wrapping is mediated by cells migrating from the implanted venous grafts into the nerve bundle.

**Methodology/Principal Findings:**

To test the hypothesis, severed femoral nerves of rats were grafted with venous grafts from animals of the opposite sex. Nerve regeneration was impaired when decellularized or irradiated venous grafts were used in comparison to untreated grafts, supporting the involvement of venous graft-derived cells in peripheral nerve repair. Donor cells bearing Y chromosomes integrated into the area of the host injured nerve and participated in remyelination and nerve regeneration. The regenerated nerve exhibited proper axonal myelination, and expressed neuronal and glial cell markers.

**Conclusions/Significance:**

These novel findings identify the mechanism by which vein wrapping promotes nerve regeneration.

## Introduction

Wrapping the scarred nerves with autogenous vein grafts has been used effectively for the treatment of peripheral neuropathy in both experimental and clinical settings [1]–[17]. In clinic, results of these studies have shown improvement of the nerve function and the symptoms associated with recurrent compressive peripheral neuropathy [5]–[10], [12], [13], [17], carpal tunnel syndrome [6], [14], tarsal tunnel syndrome [8], cubital tunnel syndrome [17], prevention of scar formation [4], [7], [11], and alleviation of painful neuroma [15], [16]. The concept that the walls of blood vessels are a reservoir of ubiquitous multi-lineage cells has largely emerged by the description of mesoangioblasts and hematopoietic stem cells [18], [19]. A vascular niche for adult hippocampal neurogenesis was identified [20] and endothelial cells were recognized as a critical component for secreting soluble factors that maintain the self-renewal and multipotency of neural stem cells [21]. More recently, adipose vasculature has been identified as a niche where white fat progenitor cells reside [22], and our publications also describe a perivascular origin for multipotent myoendothelial cells and pericytes in human skeletal muscle [23], [24].

Herein, to explore our hypothesis that the healing of peripheral nerves is mediated by vascular cells from the venous grafts, we examined the migration of the cells derived from vascular walls in nerve repair by altering the venous grafts prior to transplantation using decellularization (complete cell removal) and irradiation (preventing cell migration and proliferation) methods. Active participation of the donor vascular cells in the remyelination of the injured nerve was determined by bridging the peripheral nerve defects created in female rats using donor male venous grafts.

## Results

### Irradiation inhibits cell migration and proliferation in venous grafts

**Figure 1:**
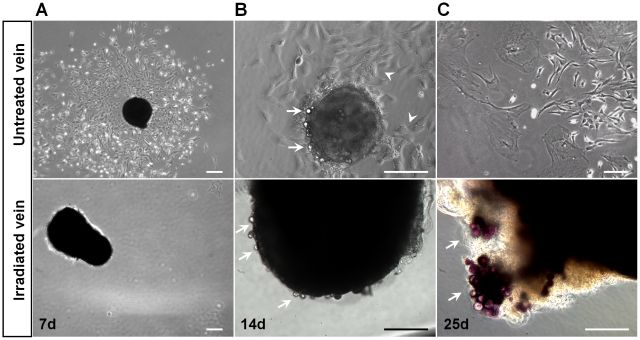
Cell migration is inhibited by irradiation. The normal untreated and irradiated veins were separately cultured on fibronectin-coated culture plates in EGM2 culture media. (**A**) Many cells migrated out of the untreated vein after 7 days in culture, while very few cells were detected in the culture dishes containing the irradiated vein. (**B**) Cells were still visible at the edges of the untreated and irradiated veins (arrows) after 14 days of culture, and continuously outgrew from the untreated veins (arrowheads). (**C**) Untreated venous graft-derived cells showed a diverse morphology; while clustered cells in the irradiated vein did not migrate out of the vein even after 25 days of culture but remained viable as verified by the MTT assay (purple). Data represent at least three independent experiments. Scale bars represent 200 µm (**A**) or 100 µm (**B** and **C**).

**Figure 2:**
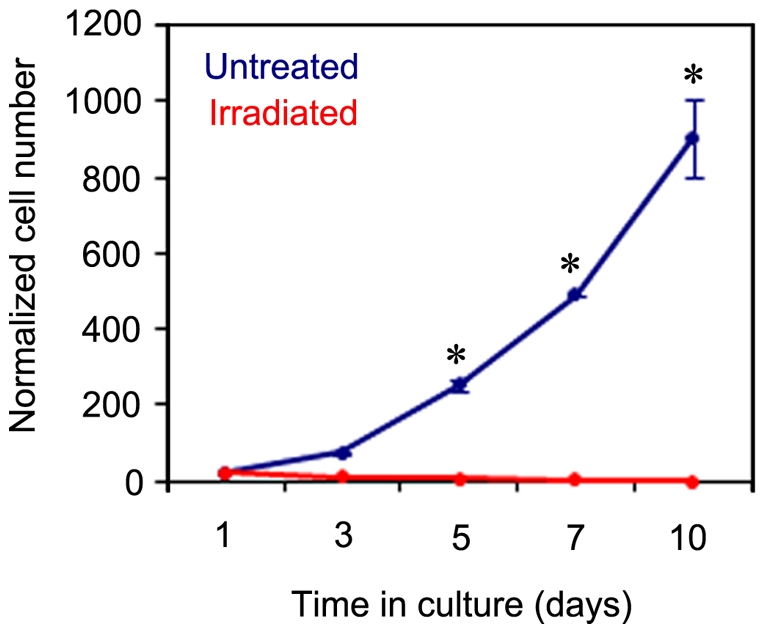
Cell proliferation is inhibited by irradiation. The viable cells from normal untreated and irradiated veins were separately cultured on fibronectin-coated culture plates in EGM2 culture media. Plotted are the normalized cell counts at each time point obtained from the analysis of 3 independent experiments per group. The cells from untreated veins showed an increased growth rate in comparison to cells from irradiated veins (*P<0.001, Student's *t*-test).

To determine the effectiveness of decellularization or irradiation on venous grafts before transplantation, parallel experiments were performed where pieces (∼1–2 mm) from untreated (control), irradiated, and decellularized (SDS-treated) veins were placed in fibronectin-coated 24-well plates in endothelial cell growth medium-2 (EGM2, n = 3). In order to optimize the irradiation dose used on the excised veins we assessed doses of 1000, 2000, 5000, and 10,000 rad. The dose of 1000-rad was chosen because it limited cell damage and preserved the integrity of the vein grafts. This radiation dose has also been shown to prevent human endothelial cell replication [25]. Upon culturing venous grafts on fibronectin-coated 24-well plates in EGM2, many cells were seen to migrate out of the normal untreated vein after 7 days in culture (**Fig. 1A**), very few cells migrated out from the irradiated veins (**Fig. 1A**), and no cells migrated from the decellularized vein explants (data not shown) as previously reported by Schaner *et al*. [26]. Cells were still visible at the edges of the untreated and irradiated veins after 14 days of culture (**Fig. 1B**, arrows), but continuously outgrew only from the untreated explants (**Fig. 1B**, arrowheads) as a mixture of progenitor cells that make up the walls of blood vessels (**Fig. 1C**). The viability of clustered cells attached to the irradiated veins was verified using the MTT [3-(4,5-dimethylthiazol-2-yl)-2,5-diphenyltetrazolium bromide] assay. Although the cells appeared to be viable (**Fig. 1C**, purple), the cells could not migrate out of the vein even after 25 days. In addition, to evaluate the proliferation of viable cells, an equivalent number of cells from untreated and irradiated veins were plated to measure their growth kinetics. Analysis of the cell number over a period of 10 days revealed that the number of cells from the untreated venous grafts significantly increased (*P*<0.001) compared to the cells from irradiated veins (**Fig. 2**). These data demonstrated that irradiation diminishes cell migration and proliferation potential.

### Cells from venous grafts contribute to nerve regeneration

**Figure 3:**
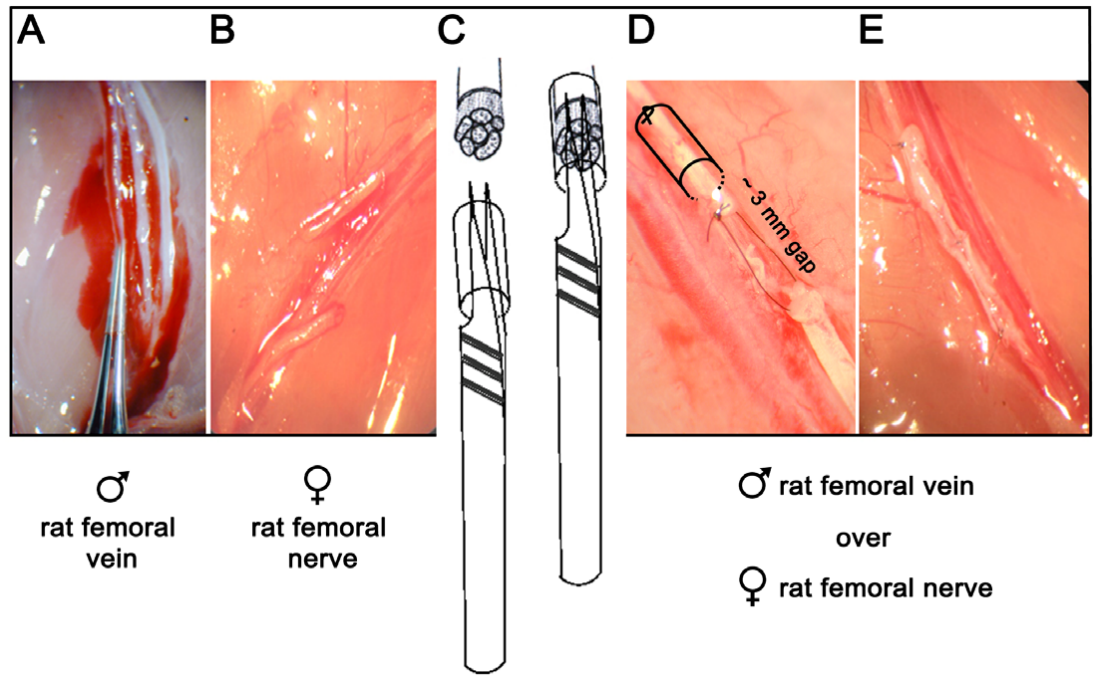
Surgical procedures for isolation and implantation of venous graft. A five to six mm segment of pure femoral vein from a male Fisher rat was isolated, pulled over a 0.2 mm forceps (vessel dilator, **A**) and placed in sterile PBS for later use. The right femoral nerve from a female rat was exposed, and a 5 mm segment of peripheral nerve was carefully separated so as not to disturb the attached artery and vein compartment. Using surgical scissors, neurotomy was performed in the middle of the femoral nerve of a female Fisher rat (**B**). The distal end of the femoral nerve was passed through the previously removed male vein graft (**C**), and cut (1 mm) to obtain a fresh end. Using 10/0 monofilament nylon, the proximal and distal stumps were drawn together and tied to form a loop while keeping a gap of ∼3 mm (**D**). Every effort was made to avoid tension and keep correct rotational alignment throughout. Using the forceps, the femoral vein was pulled over the gap toward the proximal end of the nerve. The vein graft was secured in place between ends of the femoral nerve with 10/0 monofilament nylon, using 2–3 epineurial stitching for each stump (**E**).

The role of venous graft-derived cells in the nerve repair process was determined by using venous grafts that were either irradiated or decellularized prior to their implantation. The regeneration process was then compared among the treated and untreated graft groups. To investigate whether the nerve regeneration is mediated by venous graft-derived cells, peripheral nerve lesions were bridged using venous conduits in a rat sex-mismatch model (**Fig. 3**) and fluorescence in situ hybridization (FISH) was used to detect male donor-derived Y chromosome positive nuclei in the regenerated female host nerves. Proper nerve regeneration four to six weeks after implantation was assessed using histochemistry, immunohistochemistry, and electron microscopy.

**Figure 4:**
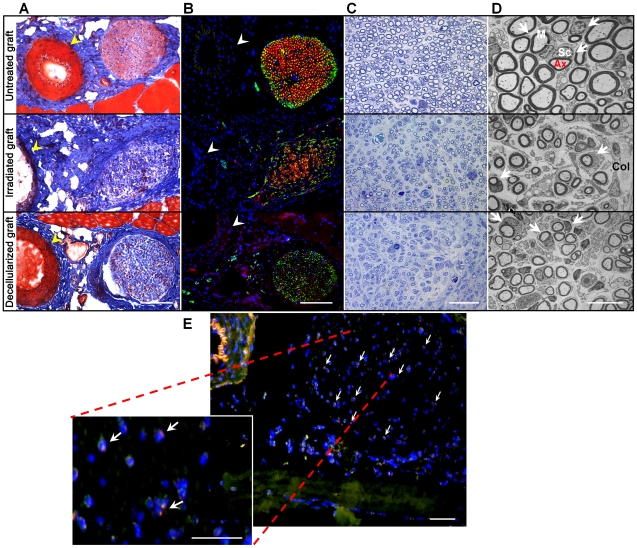
Untreated venous grafts exhibit more effective regeneration. (**A**) Eight weeks post-transplantation, Masson's Trichrome staining revealed absence of collagen matrix and adventitia of the grafted vein (blue) and defined nerve regeneration with perineurium surrounding the nerve bundles (pink) in untreated graft. Thickening of the extracellular matrix and disorganized perineurium was obvious in irradiated and decellularized grafted groups. (**B**) The cross-section of the regenerated nerve exhibited regenerated NF-positive axons (green) encompassed by FluoroMyelin-positive Schwann cells (red) suggesting proper regeneration of the femoral nerve in untreated control grafts compared to irradiated or decellularized grafts. Undamaged host artery shown with arrowheads was used as a reference point (**A, B**). (**C**) Toluidine blue staining and (**D**) transmission electron microscopy at the mid-proximal sections confirm superior regeneration with larger myelinated axons including presence of myelin-producing Schwann cells (arrows) surrounding the regenerated axons. Absence of connective tissue fibrosis was evident in untreated grafts while both irradiated and decellularized grafts showed poor levels of nerve regeneration indicated by small myelinated Schwann cells with high levels of connective tissue fibrosis marked by collagen. “Sc” corresponds to Schwann cells, “M” to myelin sheath, “Ax” to axon, and “Col” to collagen. (**E**) Four to six weeks after injury, FISH performed for the detection of Y chromosomes, verified the active contribution of male donor-derived blood vessel progenitor cells (Y chromosome-positive nuclei, red) in the regenerated female femoral nerve (host Schwann cell nuclei counterstained with DAPI), which is shown in blue (see arrows, n = 6). A total of 32 rats were transplanted (24 untreated grafts, 4 irradiated grafts, and 4 decellularized grafts). Scale bars represent 100 µm (**A–C**) or 10 µm (**D**).

At the time of harvest, the vein grafts could be identified by microscopy in all the groups without any sign of absorption or degeneration. Minimal scar or adhesions were noticed between the nerve and the vein graft, although some scarring was evident in recipients of the irradiated veins. Complete femoral nerve regeneration was observed in all of the rats that received untreated control vein grafts, while the recipients of the irradiated and decellularized veins exhibited only minimal regeneration. Six to eight weeks after transplantation, Masson's Trichrome staining of the nerves implanted with irradiated and decellularized grafts (**Fig. 4A**) showed a reduced number of myelinated axons (no traces of pink stain) and a greater amount of collagen deposition (intense blue stain), when compared to nerves with untreated grafts. The regenerated nerve in untreated venous grafts showed a well-organized architecture of myelinated nerve fibers, including less fibrotic tissue (collagen, blue) between fascicles (epineurium) and within individual axons (endoneurium) (**Fig. 4A**). Regenerated nerves in untreated grafts exhibited many neurofilament-positive axons (green) surrounded with myelin sheaths positive for FluoroMyelin (red), as compared to other experimental groups (**Fig. 4B**), suggesting proper formation of mature myelinated axons at the site of nerve regeneration. This pattern of maturation was not apparent in recipients of irradiated or decellularized veins (**Fig. 4B**). Toluidine blue staining (**Fig. 4C**), and TEM images of the same sections (**Fig. 4D**) revealed that the decellularized and irradiated veins induced only scattered regeneration in the form of small axons with thin myelin sheets and more prominent thickening of the extracellular matrix compared to recipients of untreated venous grafts (**Fig. 4C–D**).

Morphometric parameters such as the number of myelinated axons, myelin thickness, myelinated fiber area, axonal area, and g-ratio were quantitatively measured in the mid-section of the regenerated nerves using toluidine blue and TEM images (**Table 1**). Eight to ten weeks after transplantation, the number of the regenerated myelinated axons was not significantly different between the treatment groups (*P* = 0.760) when compared to untreated femoral nerves; however, there were more myelinated axons with significantly greater diameters in uninjured control nerves and nerves grafted with untreated vein grafts, compared to nerves associated with irradiated and decellularized vein grafts. Median values for myelinated fiber area and myelin thickness in irradiated and decellularized graft groups were significantly smaller than in the untreated graft group (*P*<0.05). The median axonal area and the g-ratio, which describes the relationship between axon caliber and myelinated fiber caliber, were significantly smaller in the irradiated and decellularized vein graft groups when compared to the untreated grafts and uninjured control groups (*P*<0.05). There was no significant difference seen in the median axonal area and g-ratio between the untreated graft and uninjured control nerve groups, suggesting that untreated venous grafts contributed considerably to nerve regeneration. This is in stark contrast to the irradiated and decellularized vein graft groups which failed to regain their normal axon diameter and presented with numerous regenerating fibers that were surrounded by very thin myelin sheaths when compared to the untreated graft and uninjured control groups.

**Table 1:** Morphometric analysis of the regenerated myelinated axons.

Parameters measured	Control femoral nerve	Untreated graft	Irradiated graft	Decellularized graft
Number of myelinated axons	400	375	435	440
Myelinated fiber area (µm^2^)	21.9 (11.1–28.1)	13.0^§^ (6.25–21.9)	5.25^§*^ (1.50–6.26)	3.81^§*^ (1.50–6.26)
Myelin thickness (µm)	9.72 (6.0–14.4)	5.86^§^ (3.35–8.89)	2.87^§*^ (1.84–4.57)	2.05^§*^ (0.97–3.32)
Axonal area (µm^2^)	10.1 (5.93–13.3)	7.64 (2.08–12.2)	2.19^§*^ (0.77–4.26)	1.43^§*^ (0.24–2.85)
g-ratio	0.54 (0.40–0.61)	0.50 (0.42–0.57)	0.44^§*^ (0.14–0.54)	0.36^§*^ (0.14–0.51)

The median values for the number of the myelinated axons (per 10,000 µm^2^) at the mid-proximal portion of the regenerated nerve stained with toluidine blue showed no significant difference between the groups (*P* = 0.760; ANOVA, Kruskal-Wallis on Ranks). Parametric analyses of transmission electron microscopy images of the proximal-mid cross-sections of the regenerated femoral nerve show that median (25^th^–75^th^) values of the myelinated fiber area and myelin thickness statistically differ between the groups (*P<0.05; ANOVA, Kruskal-Wallis on Ranks) when compared to the control femoral nerve (^§^ *P*<0.05; ANOVA, Kruskal-Wallis on Ranks). The quantitative measurement of the axonal area and the g-ratio (axonal area : myelinated fiber area) showed no difference between the untreated graft and control femoral nerve groups. The irradiated and decellularized groups showed significantly lower g-ratio associated with thinner axonal remyelination when compared to untreated grafts (*P<0.05, ANOVA, Kruskal-Wallis on Ranks) and uninjured control femoral nerve (^§^ *P*<0.05; ANOVA, Kruskal-Wallis on Ranks). The measured morphometric parameters represent results from 3–4 mice per group and analysis of 300–400 myelinated fibers.

To further examine if the vessel-derived donor cells play a direct role in the regeneration process, irradiated and decellularized venous grafts were used in parallel experiments along with untreated normal venous grafts. Four to six weeks after injury, FISH was performed to detect Y-chromosome bearing nuclei (red) which validated the presence of male donor-derived blood vessel progenitor cells within the regenerated female nerves of untreated venous grafts (arrows, **Fig. 4E**), but not in irradiated or decellularized grafts (data not shown). These experiments support our hypothesis demonstrating the participation of blood vessel-derived cells in the support of axonal growth and restoration after nerve injury.

## Discussion

Since most tissues are vascularized, blood vessels have been implicated as a potential source from which multipotent stem cells could originate. Indeed, multi-lineage progenitor cells have recently been found to populate blood vessel walls, notably in skeletal muscle [23], [24]. An interesting corollary to the latter findings is the fact that venous grafts have been successfully used to bridge nerve defects both experimentally and clinically [1]–[3], [6]–[17], although the biological mechanisms have remained unknown. In the present study, we put forward the hypothesis that nerve repair is perhaps mediated by the contribution of cells migrating from the implanted donor venous grafts into the injury site and have shown at least one of the biological mechanisms that contribute to the therapeutic benefits of vein wrapping. Indeed we show that, vein decellularization, which destroys the residing cells while the mechanical properties of the vessel are not impaired [27], and irradiation, that causes cell depletion and prevents cell migration and replication [25], [28] severely compromised nerve regeneration. These experiments provide evidence that without functional blood vessel-derived cell involvement, nerve regeneration mediated by vein wrapping is limited. The proper nerve regeneration and reduced endoneurial scarring seen when untreated venous grafts were utilized as the nerve guide conduit is believed to be due in part to i) the facilitation of graft invasion by increased Schwann cell motility [29], ii) the decrease in fibroblast infiltration [30], iii) the prevention of uncontrolled fiber outgrowth [31] and neuroma formation [15], [16], iv) the improvement of gliding on the smooth inner surface of the vein [9], and v) the prevention of scar tissue formation [7]. Furthermore, the venous conduits facilitate nerve regeneration through neovascularization [14] and growth factor secretion [31].

Our sex-mismatch venous graft model revealed the active participation of venous graft- derived cells in accelerating the process of remyelination and regeneration. The cells were shown to migrate from the venous nerve guide to the site of nerve injury which was evidenced by the presence of donor-derived Y chromosome possessing cells in regions of regenerated nerve. The colocalizations of these cells with host female Schwann cell nuclei reveal possible differentiation and/or fusion of vessel-derived cells to glial cells. Further experiments are necessary to reveal the identity of the donor cell types and the terminal fates of the host-derived cells. It is noteworthy that nerve guides that combine both muscle and vein have shown promising results for regenerating larger nerve defects [32], [33]. Clinical applications that have used combined muscle-vein grafting have led to efficient nerve repair, both for motor and sensory nerves, with satisfactory functional recovery in 85% of the patients [32]. We suggest that the success of these muscle-vein grafts is due to the cells that are contained therein including satellite cells (and other muscle progenitor cells), myoendothelial cells, and pericytes. These cells could then differentiate toward a glial lineage and/or secrete exogenous trophic factors reinforcing the myelination and regeneration in a paracrine manner.

This study demonstrated a key biological mechanism behind the successful use of vein wrapping for nerve repair. The improved axonal in-growth was shown to occur through cellular interactions between the migrating cells emanating from the venous grafts and the regenerating nerve stumps.

## Materials and Methods

### Vein decellularization and irradiation procedure

With the aid of an operating microscope (WILD M690; WILD HEERBRUGG, Switzerland), segments of pure femoral veins were isolated and decellularized according to a protocol previously reported by Shaner *et al*. [26]. Briefly, isolated femoral veins were placed into 0.075% sodium dodecyl sulfate (SDS; Bio-Rad Laboratories) in PBS for 15 h at 37°C in a shaking water bath. Subsequently, they were rinsed by agitation in PBS at 37°C for 15 min repeatedly for five times. This method of decellularization has been confirmed to remove >94% of cells from the vein. For irradiation study, the vein grafts were irradiated at 1000-rad (4.4 min) dose.

### MTT assay

The MTT [3-(4,5-dimethylthiazol-2-yl)-2,5-diphenyltetrazolium bromide] assay was carried out to evaluate cell viability [34]. Sterile solution of MTT (5 mg/ml stock; Sigma) was added to the culture dishes (0.1 ml/ml media) containing control and experimental venous grafts, and the dishes were incubated for 30–60 min at 37°C. Mitochondrial dehydrogenase of viable cells cleaves the tetrazolium rings of the pale yellow MTT and form purple formazan crystals which are impermeable to cell membranes, thus resulting in its accumulation within healthy cells. Presence of purple color was detected and captured using a Leica DMIRB microscope equipped with a Retiga 1300 digital camera (Q imaging) and Northern Eclipse software system (v. 6.0; Empix Imaging, Inc.).

### Cell proliferation

An equivalent number of viable cells were plated in fibronectin-coated 24-well plates in endothelial cell growth media-2 (EGM2; Lonza). The cell number in each group was counted with a hemocytometer and standard trypan blue technique for cell viability determination over a 10-day period from 3 independent experiments.

### Isolation and implantation of venous nerve guide

All animal experimentation including the surgical procedures illustrated in Figure 2 were approved by the University of Pittsburgh Institutional Animal care and use committee (Animal Welfare Assurance Number A3187-01) under approved current protocol #0810153A-2 (Formerly Protocol #13-03). Female Fischer 344 rats weighing 100–125 grams and male Fischer 344 rats weighing 200–225 grams were obtained from Harlan (Indianapolis, IN). Rats were initially anaesthetized using 4% isoflurane (VETEQUIP Inc., CA) in O_2_ and maintained under anesthesia using 1.5% isoflurane in O_2_. Under sterile conditions, with the aid of an operating microscope (WILD M690; WILD HEERBRUGG, Switzerland), a 5 to 6 mm segment of pure femoral vein from a male rat was isolated, pulled over a 0.2 mm forceps (vessel dilator), and placed in sterile saline solution for later use. The right femoral nerve of a female rat was exposed through anterior approach, and a 5 mm segment of peripheral nerve was carefully separated so as not to disturb the attached artery and vein compartment. Using surgical scissors, neurotomy was performed in the middle of the femoral nerve. The distal end of the nerve was passed through the previously removed male vein graft, and cut (1 mm) to obtain a fresh end. Using a 10/0 monofilament suture, the proximal and distal stumps were connected together and the nylon was used to form a loop while keeping a gap of ∼3–4 mm. Every effort was made to avoid tension and keep correct rotational alignment throughout. Using the forceps, the femoral vein was pulled over the gap toward the proximal end of the nerve. The vein graft was secured in place between ends of the femoral nerve with 10/0 monofilament nylon, using 2–3 epineurial stitching for each stump. A small knot was placed with a 4/0 silk suture on the fascia of the muscle above the construct at the proximal and distal end to allow easy identification at the time of harvest and cryosectioning. Rats were monitored daily post-surgery for proper recovery to assess and alleviate any signs of pain and distress. A total of 32 rats received vein grafts for the sex-mismatched study (24 untreated grafts, 4 irradiated grafts, and 4 decellularized grafts) and 3 rats were used for sex-matched study as a negative control for FISH analysis.

### Histochemistry

For Masson's Trichrome stain, the slides were processed as detailed in the manufacturer's protocol (Masson's Trichrome stain kit, K7228; IMEB Inc.), which stains collagen blue, muscle fibers red, and nuclei black.

### Immunohistochemistry

Cryosections of the reconstructed femoral nerves were first immunostained for FluoroMyelin Red (Molecular Probes) according to the protocol provided by the manufacturer, and then fixed in 4% PFA, blocked with 5% donkey serum for 1 h, and incubated with neurofilament (NF, 1∶300, rabbit; Chemicon) antibody for 2 h at room temperature. Tissues were then rinsed with PBS and stained with secondary antibody AlexaFluor® 488-conjugated for 20 min. Nuclei were revealed with DAPI. Fluorescent images were captured and processed using a Nikon Eclipse E800 microscope equipped with a Q imaging Retiga Exi digital camera using Northern Eclipse software (v. 6.0; Empix Imaging Inc.).

### Morphometric analysis of the regenerated nerve

To evaluate the degree of myelination, some of the reconstructed femoral nerves including all tissue surrounding them, were processed for light and transmission electron microscopy. The contralateral sciatic nerves harvested at the same level served as a “normal” control. Briefly, the tissues were fixed in 2.5% glutaraldehyde (EM grade; Taab Chemical) in 0.1 M phosphate buffer (pH 7.3; Fisher Scientific) overnight at 4°C. The nerves were then rinsed in PBS, post-fixed in 1% osmium tetroxide (Electron Microscopy Sciences) with 0.1% potassium ferricyanide (Fisher Scientific), dehydrated in a series of ethanol solutions (30%–90%; Fisher, and 100% [Ethanol 200 Proof]; Pharmco), exposed to propylene oxide twice for 10 min each and embedded in Epon (Dodecenyl Succinic Anhydride, Nadic Methyl Anhydride, Scipoxy 812 Resin and Dimethylaminomethyl; Energy Beam Sciences). All grafted segments were carefully oriented in order to obtain sections perpendicular to their long axis. Semi-thin (300 nm) sections were cut using a Leica Ultracut and stained with 0.5% toluidine blue (Fisher Scientific) and examined under a light microscope (Olympus BX51) with Magnafire 2.1A image capture software, and quantified for the number of myelinated axons. The ultrathin sections (65 nm) were obtained at distal, mid, and proximal ends using a Leica Ultracut Microtome and stained with 2% uranyl acetate (Electron Microscopy Sciences) and Reynold's lead citrate (Fisher Scientific), and examined on a JEOL JEM-1011 transmission electron microscope. Each nerve section was divided into four quarters and each quarter was analyzed individually. Morphometric parameters of 300–400 fibers were calculated following image capture, background subtraction, image enhancement, automatic thresholding, and final editing. The total number of myelinated axons was counted in ∼10,000 µm^2^ of the cross-sectional area from the mid-sections of the nerve, and median area of myelin thickness, median cross-sectional area of myelinated axons, and g-ratio (axonal area/myelinated fiber area) were examined.

### Fluorescence in Situ Hybridization (FISH)

Venous graft cryosections (6 µm) were fixed in methanol : acetic acid (3∶1), dehydrated using 70, 80, 95, and 100% ethanol for 3 min each, air dried, and then denatured in 70% formamide-2× sodium chloride-sodium citrate (SSC) for 5 min at 69±1°C. Slides were then dehydrated as described above and air dried. Following manufacture's protocol, we applied the directly-labeled fluorescence rat-specific Y-chromosome-Cy3 and chromosome 12-FITC probes (Cambio) to the target areas (area around the sections marked with diamond scribe). The slides were covered with plastic coverslips (VWR) then glued with rubber cement and incubated in a humidity chamber for 18–24 h at 37°C. The next day, rubber cement and coverslips were removed and the slides were washed with 2× SSC for 5 min, followed by washes in 50% formamide-2× SSC (12–13 min), 2× SSC (three times for 7 min) and 2× SSC-Tween (2 min). All washes were done in 45°C. Slides were counterstained with DAPI and were mounted with Vectashield® medium (Vector). The hybridized Y-chromosome-specific probe was detected using a fluorescence microscope. Negative control tissues were consisted of sex-matched venous grafts (female femoral vein transplanted over the female femoral nerve) to check for absence of the Y-chromosomes signals.

### Statistics

All Statistical analyses were carried out using SigmaStat (Jandel Scientific v2.0) software package. The Student's *t*-test or Mann-Whitney Rank Sum test was used for direct comparisons between treated and untreated groups. For multiple comparisons, One-way ANOVA or the Kruskal-Wallis One-way ANOVA on ranks (where appropriate) was applied. A *P* value<0.05 was considered significant. Morphometric parameters are expressed as the median values, 25^th^–75^th^ percentiles.
